# Use of general practice, diagnostic investigations and hospital services before and after cancer diagnosis - a population-based nationwide registry study of 127,000 incident adult cancer patients

**DOI:** 10.1186/1472-6963-12-224

**Published:** 2012-07-28

**Authors:** Karina Garnier Christensen, Morten Fenger-Grøn, Kaare Rud Flarup, Peter Vedsted

**Affiliations:** 1 The Research Unit for General Practice, Research Centre for Cancer Diagnosis in Primary Care (CaP), Aarhus University, Bartholins Allé 2, 8000, Aarhus C, Denmark

**Keywords:** Cancer, General practice, Diagnostic, Hospital admission, Outpatient visit, Health services, Consultation, Registry study, Aftercare, Denmark

## Abstract

**Background:**

Knowledge of patterns in cancer patients’ health care utilisation around the time of diagnosis may guide health care resource allocation and provide important insights into this groups’ demand for health care services. The health care need of patients with comorbid conditions far exceeds the oncology capacity and it is therefore important to elucidate the role of both primary and secondary care. The aim of this paper is to describe the use of health care services amongst incident cancer patients in Denmark one year before and one year after cancer diagnosis.

**Methods:**

The present study is a national population-based case–control (1:10) registry study. All incident cancer patients (n = 127,210) diagnosed between 2001 and 2006 aged 40 years or older were identified in the Danish Cancer Registry. Data from national health registries were provided for all cancer patients and for 1,272,100 controls. Monthly consultation frequencies, monthly proportions of persons receiving health services and three-month incidence rate ratios for one year before and one year after the cancer diagnosis were calculated. Data were analysed separately for women and men.

**Results:**

Three months before their diagnosis, cancer patients had twice as many general practitioner (GP) consultations, ten to eleven times more diagnostic investigations and five times more hospital contacts than the reference population. The demand for GP services peaked one month before diagnosis, the demand for diagnostic investigations one month after diagnosis and the number of hospital contacts three months after diagnosis. The proportion of cancer patients receiving each of these three types of health services remained more than 10% above that of the reference population from two months before diagnosis until the end of the study period.

**Conclusions:**

Cancer patients’ health service utilisation rose dramatically three months before their diagnosis. This increase applied to all services in general throughout the first year after diagnosis and to the patients’ use of hospital contacts in particular. Cancer patients’ heightened demand for GP services one year after their diagnosis highlights the importance of close coordination and communication between the primary and the secondary healthcare sector.

## Background

One main task of a well-organised healthcare system is to effectively diagnose and treat serious diseases in a way that optimises the prognosis. Studies on the diagnosis of cancer suggest that non-conclusive initial visits and a long waiting period for investigations to be performed are likely to delay the diagnosis [1,2] which may have a negative effect on survival [3,4]. Up to 2020, we expect to see a 20% increase in the incidence of cancer; a growth that may be attributed to demographic changes and advances in medicine which means that more citizens will be living with cancer [5]. In this context, more knowledge of cancer patients’ patterns of demand for health care services before as well as after their diagnosis is critical to identifying possibilities for improving both patient pathways and health care resource allocation.

A major concern in Denmark is that cancer patients have a poorer survival than patients in other European countries [6]. This has partly been explained by delay in cancer diagnosis and treatment [3,4,7]. More detailed knowledge of cancer patients’ health care resource demands and utilization patterns around the time of diagnosis may undoubtedly allow us to better organize health care supply and further shorten the time to cancer diagnosis, notably if particular health care utilizations patterns can be identified in large population-based cohorts. Data from such studies may prove even more valuable if combined with information on referral to diagnostic investigations and use of hospital services which would help us identify specific patterns of health care use rooted in current clinical or organizational inexpediencies. Such research would also serve the purpose of further substantiating or qualifying previous research. Apart from a comprehensive British survey which showed much variation in the number of consultations with cancer symptoms before hospital referral for suspected cancer [8], most previous studies have included fewer than 500 cancer patients and have suggested that before the time of diagnosis, cancer patients use their general practitioner (GP) less than controls [9,10] with GP consultation frequencies peaking in the first month after diagnosis [11].

Once a cancer patient has been diagnosed and treatment has been initiated, cancer trajectories are very different. However, common for all cancers is the claimed lack of primary care involvement after discharge, which may be ascribed to patients being reluctant to go back to primary care and primary care not being there for the patients [12-16]. We therefore need a precise description of cancer patients’ health care usage in primary and secondary care after their cancer diagnosis. Such detailed knowledge would be particularly instrumental in identifying their need for health care services in the period after discharge from hospital.

The aim of this study was to describe incident adult cancer patients’ health service utilisation one year prior to and one year after their first cancer diagnosis.

## Methods

### Study design and setting

The present study was a population-based case–control registry study with a 1:10 age and gender matching. Data on health service utilisation were collected for a period spanning from one year before to one year after the date of cancer diagnosis. The date of diagnosis was extracted from the Danish Cancer Registry [17].

In Denmark, health care services are free and tax-financed. Nearly all citizens (98%) are registered with a particular general practice. GPs act as gatekeepers to most of the remaining health care providers and most cancer-specific investigations are performed in public hospitals after referral. Some diagnostic investigations (ultrasound and conventional x-ray), endoscopies and biopsies can also be made by private practising specialists. Although cancer treatment takes place in public hospitals which are in charge of the cancer patient’s treatment until he or she is discharged, the cancer patient needs continuous primary health care and cooperation between the primary and the secondary sector is a cornerstone in a comprehensive, patient-centred approach. Furthermore, the number of patients with comorbid conditions and their health care need far exceed the oncology capacity. It is therefore important to establish knowledge on the present role of primary care.

### Study population

Denmark operates comprehensive population-based registries that link information on each citizen by a unique personal registration number assigned to every Danish citizen upon birth. Using the Danish Cancer Registry launched in 1943, we identified those patients who were diagnosed with a malignant neoplasm with ICD10 codes C00 to C97, except C44 (non-malignant melanoma), between 1 January 2001 through 31 December 2006 and who had not been registered with any previous notifiable cancer diseases from 1943 onwards (the abovementioned ICD10 codes and B21, D06, D07.6, D09.0, D09.1, D30, D32, D33, D35.2, D35.3, D35.4, D37 - DD48, E34, N87, and O01) [18]. Patients who were 40 years or older were included, because at all ages women have higher GP consultation rates than men with a peak between ages 15 and 35 [19] which could have biased the data. We thereby captured more than 95% of all incident cancers. Until 1978, the Danish Cancer Registry did not contain ICD10 codes, but solely ICD7 codes, and 120 patients were therefore excluded because they had such previous ICD7-coded cancer diagnoses. Only patients registered with a date of birth and gender in the Central Population Registry were included. Patients were excluded if they moved to another country during the observation period around the date of diagnosis, or if they got a second cancer (except for metastases with ICD10 codes C76-C80 and recurrent cancers in the same organ) in the two-year period after their incident cancer diagnosis.

Using incidence density sampling [20], we matched each cancer patient on gender and date of birth with ten controls from a reference population not registered with a cancer diagnosis in the Danish Cancer Registry until two years after the index date. The index date was defined as the date of diagnosis of the case. Persons born in 1930 or before were age-matched on the year of their birth because the groups were small. Controls could be sampled as controls more than once for different cases, but only once for the same case. The use of incidence density sampling meant that a control could also later be included as a cancer case (after two years).

### Registry data

Data regarding date and type of cancer diagnoses were retrieved from the Danish Cancer Registry. Statistics Denmark conducted data linkage to the National Health Insurance Service Registry, the National Patient Discharge Registry, the Central Population Registry, the Registry of Causes of Death as well as to sociodemographic and socioeconomic variables which were also provided by Statistics Denmark. Personal registration numbers were pseudomised by Statistics Denmark which hosted the data. Approval was obtained from the Danish Data Protection Agency (journal no. 2009-41-3471), whereas approval from the Danish Ethical Committee is not required for registry studies.

Data on primary and secondary health service utilisation were collected from 1 January 2000 through 31 December 2007. The study period spanned the period from one year before to one year after the cancer diagnosis. Data from the National Health Insurance Service Registry included the number of face-to-face consultations in general practice in daytime and out-of-hours including home visits. Data regarding diagnostic investigations comprised x-ray (performed by radiologists), ultrasound (performed by gynaecologists and surgeons), endoscopies (performed by otorhinolaryngologists, gynaecologists, internists, surgeons and GPs) and biopsies (performed by otorhinolaryngologists, ophthalmologists, dermatologists, gynaecologists, internists, surgeons, orthopaedic surgeons and GPs). Data from the National Patient Discharge Registry gave the number of somatic hospital admissions, outpatient visits and diagnostic investigations (x-ray, ultrasound, CAT scan, MRI scan, angiography, endoscopies and biopsies). Contacts for both discharged and non-discharged outpatients were included. Endoscopies included all endoscopies performed through natural body orifices only. Biopsies comprised all procedure codes containing the word biopsy in the descriptive text. For all variables from the National Patient Discharge Registry, only one event per category was included per day due to the complexity of the registrations in this registry. Age, gender and country of residence were obtained from the Central Population Registry, while the date of death was obtained from the Registry of Causes of Death.

The demographic and socioeconomic variables included country of origin, marital status, taxable income using the OECD-modified scale [21], highest attained education categorised according to the International Standard Classification of Education (ISCED) [22], and labour market affiliation. Data were retrieved for the year of the diagnosis or index date, except for country of origin where the latest registered value was selected due to inconsistencies in the registrations. See Table 1 for definition and categorisation of these variables.

**Table 1:** Categorisation and definition of sociodemographic and socioeconomic indicators

**Demographic and socioeconomic indicators**	**Categorisation**	**Definition**
Age	40-59 years	Age was calculated at the day of diagnosis for the cancer patients. Controls were matched on age and were thus in the same age group as their respective cases.
	60-79 years	
	>80 years	
Country of origin	Danish	Western countries are defined as: Nordic countries, European Union countries, Andorra, Liechtenstein, Monaco, San Marino, Switzerland, the Vatican State, Canada, USA, Australia, New Zealand. Non-Western countries are all remaining countries.
	Immigrant/descendant Western	
	Immigrant/descendant non-Western	
Marital status	Married	Married are persons living in civil union or being married. Remaining persons belong to the not married category.
	Not married	
Education	Basic	Highest attained education categorised according to the International Standard Classification of Education (ISCED). Basic: Primary and lower secondary, 0–10 years.
	Short	Short: Upper secondary and post-secondary non-tertiary, 11–15 years.
	Long	Long: Tertiary and advanced research programmes, >15 years.
	Unknown	
Labour market affiliation	Working	Based on main employment during the preceding 12 months.
	Unemployed	
	Retired	
	Leave and other	
Income	Lowest 20%	Taxable income during the preceding 12 months using the OECD-modified scale.
	Middle 50%	
	Highest 30%	

### Outcome variables

The outcome measure was the incidence rate of health services received per month and per three months one year before and one year after diagnosis. The index date (date of cases’ cancer diagnoses) was contained in the month before the diagnosis. The proportion of persons receiving health services was calculated per month. Health services were collated into three groups: GP face-to-face consultations (daytime and out-of-hours), diagnostic investigations (primary and secondary sector) and hospital contacts.

### Analysis

The date of consultation provided by the National Health Insurance Service Registry was given as a week number which was converted into a date in order to be able to calculate the interval from the diagnostic date to the date at which the health care service was provided. A negative binomial model was applied for the calculation of estimates and corresponding 95% confidence intervals for monthly and three-month incidence rates and rate ratios between cancer patients and the reference population of GP consultations, diagnostic investigations and hospital contacts. Robust variance estimation with clustering on patient level was used. To account for differences in follow-up time (relevant after diagnosis only), log-transformed risk time was included in the model with the regression parameter restricted to 1. Censoring was thus done for all persons one year after the index date (date of diagnosis for cases) or when a person died, whichever came first. Separate analyses were performed for females and males, as gender-specific cancers represented 21% and 12% for women and men, respectively, and because it is known that men and women differ in their use of health care services [19]. Stata 12 was used for all analyses.

## Results

The study included a total of 127,210 cancer patients and 1,272,100 age and gender-matched controls. Among cancer patients, 49.8% were women and 50.2% were men; the age group 60–79 years represented 50.4% of the women and 60.6% of the men (Table 2). Table 2 shows that the characteristics of the cancer patients and the reference population were similar with respect to country of origin, marital status, education, labour market affiliation and income.

**Table 2:** Characteristics of the reference population and the cancer patients

	**Women**				**Men**			
	**References**		**Patients**		**References**		**Patients**	
**Total (n (%))**	633,620	100.0	63,362	100.0	638,480	100.0	63,848	100.0
**Age (years)**								
40-59	193,210	30.5	19,321	30.5	144,390	22.6	14,439	22.6
60-79	319,440	50.4	31,944	50.4	387,190	60.6	38,719	60.6
80+	120,970	19.1	12,097	19.1	106,900	16.7	10,690	16.7
**Country of origin**								
Danish	603,303	95.2	60,881	96.1	612,551	95.9	61,698	96.6
Immigrant/descendant Western country	17,723	2.8	1,652	2.6	13,396	2.1	1,281	2.0
Immigrant/descendant non-Western country	12,444	2.0	829	1.3	12,308	1.9	867	1.4
**Marital status**								
Married	322,777	50.9	30,971	48.9	435,587	68.2	42,116	66.0
Not married	310,843	49.1	32,391	51.1	202,893	31.8	21,732	34.0
**Education**								
Basic	279,425	44.1	28,461	44.9	228,450	35.8	23,319	36.5
Short	185,337	29.3	18,624	29.4	252,794	39.6	25,580	40.1
Long	81,025	12.8	7,777	12.3	84,125	13.2	7,640	12.0
Unknown	87,833	13.9	8,500	13.4	73,111	11.5	7,309	11.4
**Labour market affiliation**								
Working	185,897	29.3	17,886	28.2	196,051	30.7	17,741	27.8
Unemployed	19,256	3.0	1,972	3.1	13,234	2.1	1,536	2.4
Retired	419,395	66.2	42,636	67.3	424,694	66.5	44,043	69.0
Leave and other	8,898	1.4	868	1.4	4,255	0.7	522	0.8
**Income**								
Low	130,388	20.6	13,129	20.7	123,343	19.3	12,916	20.2
Middle	320,401	50.6	32,355	51.1	314,205	49.2	32,491	50.9
High	182,661	28.8	17,878	28.2	200,693	31.4	18,438	28.9

Notes: The references (1:10) were matched on age and gender.

Missing data are not showed when representing less than 1%.

### Before the cancer diagnosis

**Figure 1:**
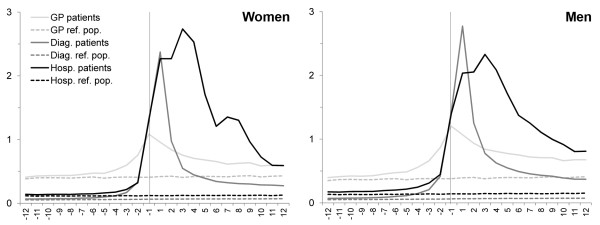
**Incidence rates of health services received per month by cancer patients and reference population.** Cancer patients (n = 63,362 women and 63,848 men). Reference population (n = 633,620 women and 638,480 men). Incidence rates were adjusted for time at risk. Vertical line indicates date of diagnosis. GP: General Practitioner; Diag.: Diagnostic investigations; Hosp.: Hospital contacts

Figure 1 shows the monthly incidence rates for use of general practice, diagnostic investigations and hospital contacts for cancer patients and the reference population divided into women and men. GP consultation patterns changed from a modest rise in both genders five to six months before diagnosis to a steep rise that peaked around one month before diagnosis. The number of diagnostic examinations and hospital contacts started to rise three to four months before diagnosis with a steep rise setting in two months before diagnosis.

**Figure 2:**
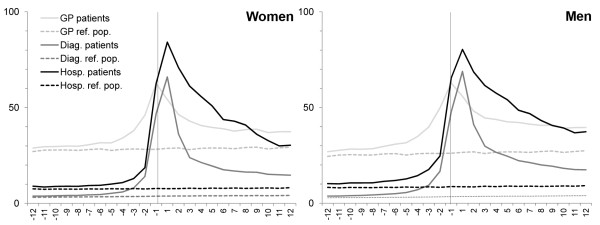
**Percentage of cancer patients and reference population receiving health services per month.** Cancer patients (n = 63,362 women and 63,848 men). Reference population (n = 633,620 women and 638,480 men). The proportion was adjusted for time at risk. Vertical line indicates date of diagnosis. GP: General Practitioner; Diag.: Diagnostic investigations; Hosp.: Hospital contacts

Figure 2 shows the proportions of persons being in contact with the healthcare system each month. The same pattern was seen as for the monthly incidence rates. In the month up to the diagnosis, approx. 60% of the cancer patients were seen in general practice compared with approx. 25% of the reference population. Even more pronounced differences were found for hospital services and diagnostic investigations. Throughout the whole period before the diagnosis, cancer patients used more health care services than the reference population and the six months preceding their diagnosis saw a steep rise in their consumption of health care services (Table 3).

**Table 3:** Incidence rate ratios for three-monthly use of general practice, diagnostic investigations and hospital services

		**Women**		**Men**	
**Months from diagnosis**	**Services**	**IRR**	**95% CI**	**IRR**	**95% CI**
−12 to −10	GP	1.07	1.06-1.09	1.13	1.12-1.15
	Diagnostics	1.24	1.20-1.28	1.37	1.32-1.42
	Hospital	1.21	1.18-1.25	1.31	1.28-1.35
−9 to −7	GP	1.10	1.09-1.11	1.17	1.16-1.19
	Diagnostics	1.32	1.28-1.36	1.51	1.46-1.56
	Hospital	1.24	1.21-1.28	1.38	1.35-1.42
−6 to −4	GP	1.20	1.19-1.21	1.34	1.33-1.36
	Diagnostics	1.66	1.61-1.71	2.08	2.02-2.15
	Hospital	1.39	1.35-1.43	1.64	1.60-1.68
−3 to −1	GP	1.97	1.96-1.99	2.38	2.36-2.39
	Diagnostics	9.61	9.47-9.76	10.86	10.69-11.03
	Hospital	5.27	5.20-5.34	5.16	5.09-5.23
1 to 3	GP	2.12	2.10-2.14	2.54	2.51-2.56
	Diagnostics	24.49	24.11-24.87	29.20	28.73-29.67
	Hospital	19.91	19.68-20.14	14.91	14.73-15.10
4 to 6	GP	1.67	1.65-1.69	2.05	2.02-2.07
	Diagnostics	6.25	6.12-6.38	8.46	8.29-8.64
	Hospital	15.11	14.89-15.34	12.08	11.90-12.27
7 to 9	GP	1.50	1.48-1.52	1.84	1.81-1.86
	Diagnostics	4.88	4.77-4.99	6.42	6.27-6.57
	Hospital	9.98	9.80-10.16	7.86	7.72-8.00
10 to 12	GP	1.43	1.41-1.45	1.73	1.70-1.75
	Diagnostics	4.37	4.27-4.48	5.49	5.35-5.63
	Hospital	5.29	5.19-5.40	5.82	5.71-5.94

Notes: IRR: Incidence rate ratio.

95% CI: 95% Confidence interval.

GP: Face-to-face contacts with GPs in both daytime and out-of-hours.

Diagnostics: Diagnostic investigations including radiological investigations, ultrasound, endoscopies and biopsies.

Hospital: Admissions and outpatient visits.

n (women) = 63,362 cancer patients and 633,620 controls.

n (men) = 63,848 cancer patients and 638,480 controls.

### After the cancer diagnosis

After having received their diagnosis, cancer patients used more health care services than their controls after adjusting for death. Figure 1 shows a marked use of hospital services in the year after the diagnosis; yet, the use of diagnostic investigations, in particular, fell rapidly. The increased monthly use of general practice flattened off around six months after diagnosis; and one year after diagnosis, the GP incidence rate was at the same level as for hospital services. Gender-specific consultation patterns were observed: men had more diagnostic investigations than women; whereas women had more hospital contacts than men. As seen in Figure 2, more than 80% of the cancer patients received hospital services in the month after their diagnosis compared with less than 10% of the controls. The proportion of cancer patients receiving each of the three types of health services remained more than 10% above that for the controls from two months before diagnosis until the end of the study period (Figure 2). Twelve months after diagnosis, the proportion of cancer patients being in contact with GPs on a monthly basis was approx. 40% - which is slightly higher than the proportion of patients having hospital contacts.

Cancer patients’ propensity to be in contact with general practice remained high: they had 43-73% more GP consultations than the reference population 12 months after their diagnosis (Table 3); and this trend was even more pronounced for the use of diagnostic investigations and hospital services in the entire year after their diagnosis.

## Discussion

### Main findings

During the six months leading up to diagnosis, Danish cancer patients started using more primary and secondary health care services than the reference population. It came as no surprise that they were also much more prone to be in contact with the health care system in the aftercare period. The timing of the peaks of use of specific services indicates that for cancer patients as a group there is a time interval of some months between patients start attending general practice and the diagnosis.

### Strengths and weaknesses

A strength of the present study is that we included a whole nation’s incident cancer patients. This gives the present study a high degree of statistical precision. Another strength is that Danish health service registries are known to be valid [23,24] because of the completeness of the registration of the Danish population and the continuity of registrations. The case–control study design was used to allow us to portray the baseline use of health care services of a reference population comparable with the cancer patients. The characteristics of the two populations were very much alike, which represents a third strength of the present study.

A weakness is that the date of diagnosis registered in the Danish Cancer Registry was systematically set to the first day in the month until 1 January 2004 where exact dates were introduced. Weaknesses in the validity that may arise because of changes in the definitions of variables over time, changes in coding or data entry procedures did not seem to affect the data as the control group’s use of health care services remained stable during the study period. We included all types of cancer as the study overall illustrates how cancer is treated within a healthcare system. Thus, differences between specific cancer types and groups of patients will undoubtedly exist as shown in a recently published British survey including 41,299 cancer patients [8]. Further research should investigate this while including the time period of the consultations.

### Comparisons with other studies

The aforementioned comprehensive British survey found that women were more likely than men to have had three or more GP consultations before hospital referral [8]. In the present study, we found a similar GP consultation pattern for both genders prior to diagnosis. Aside from the British survey [8], few existing studies have elucidated cancer patients’ health service utilisation around the time of diagnosis and they all studied fewer than 500 patients. Moreover, their methods, focus and results differed which makes direct comparison difficult. A Dutch breast cancer study using GP records found that the percentage of women seeing their GP peaked at 90% in the first month after diagnosis [11]; contrary to this, our study showed that the percentage of cancer patients seeing their GP peaked in the month up to diagnosis at 63% for both men and women. A questionnaire study on delay in the diagnosis of colorectal cancer found that patients with a severe diagnostic delay had 2.5 consultations before the disease was diagnosed compared with 1.3 visits among those patients without severe delay [25], but no information about the timing of the visits was given. An interview study combined with data from hospital records of gastrointestinal cancer patients found a mean interval of 10 weeks between GP consultation and hospital referral [26], while we found a time interval of some months between the start of attending general practice and the start of treatment for cancer patients as a group. In our study, the first observed peak was in GP consultations, which is in accordance with previous studies which have found that cancer patients first contact their GPs [16,27-29].

### Implications for future research

The present study fills major gaps in current knowledge about cancer patients’ health service utilisation around the date of diagnosis and it hence identifies targets for organizational improvement and informs a future research agenda in this field. As previously suggested, the GPs seem to play an essential role in initiating cancer diagnosis [27,30]. One way of shortening the diagnostic interval could be to reduce the number of non-conclusive GP consultations by facilitating GPs access to fast and relevant diagnostic investigations, and by optimizing the hospital-based treatment phase. We saw that the health care utilisation pattern started changing six months prior to the cancer diagnosis. Future studies should elucidate this period with regards to e.g. different cancer types, the specific health services given by the different providers and demographic and socioeconomic patient characteristics. Such studies may help identify clinical and organizational inexpediencies, which is critical to optimal health care resource allocation and, not least, to patient pathway optimization.

The present study shows that 12 months after diagnosis, primary care was, indeed, involved in aftercare as was the hospital sector. The claimed lack of primary care involvement after discharge could perhaps originate in the lack of clear communication regarding task distribution as pointed out in a study on palliative home care for cancer patients [31]. Future research should investigate the organisation of aftercare in general and the transition between primary and secondary care in particular.

## Conclusions

In cancer patients’ pathway, the diagnostic window seems to open several months before the diagnosis is made as evidenced by a rising number of GP consultations, diagnostic examinations and hospital contacts. Whether this pattern of health care use is a sign of insufficient clinical or organisational knowledge, this study cannot answer. However, there seems to be a possibility of reducing the time from GP consultation to diagnostic investigations and treatment. During the period after diagnosis, the use of all health care services remained increased with hospital contacts being most prevalent. Contacts with general practice were also increased during the first year after diagnosis which documents the importance of coordination and planning of cancers patients’ post-treatment course to improve survival. Future studies must be performed as detailed studies of specific health care services provided to specific types of cancer patients and their appropriateness in relation to effectiveness and equity in order to optimise the delivery of health services.

## Abbreviations

GP: General practitioner; ICD10: International Classification of Diseases version 10; ICD7: International Classification of Diseases version 7; CAT scan: Computed axial tomography scan; MRI scan: Magnetic resonance imaging scan; OECD: Organisation for Economic Co-operation and Development; ISCED: International Standard Classification of Education; IRR: Incidence rate ratio; 95% CI: 95% confidence interval.

## Competing interests

The authors declare that they have no competing interests.

## Authors’ contributions

PV and KGC conceived the study. The data collection and analysis were done by KGC in consultation with KRF and PV. KGC performed the statistical analyses in consultation with MFG. KGC drafted the manuscript and all authors contributed to critically revising the paper. Finally, all authors read and approved the submitted manuscript.

## Authors’ information

Research Unit for General Practice and Research Centre for Cancer Diagnosis in Primary Care (CaP), Aarhus University, Bartholins Allé 2, 8000 Aarhus C, Denmark.

## Pre-publication history

The pre-publication history for this paper can be accessed here:

http://www.biomedcentral.com/1472-6963/12/224/prepub
